# Loss of Circadian Timing Disrupts Theta Episodes during Object Exploration

**DOI:** 10.3390/clockssleep2040038

**Published:** 2020-12-01

**Authors:** Adrienne C. Loewke, Alex Garrett, Athreya Steiger, Nathan Fisher, H. Craig Heller, Damien Colas, Norman F. Ruby

**Affiliations:** Biology Department, Stanford University, Stanford, CA 94305, USA; Adrienne.Loewke@ucsf.edu (A.C.L.); alex.su.garrett@gmail.com (A.G.); athreyasteiger@alumni.stanford.edu (A.S.); nate.jfisher@gmail.com (N.F.); hcheller@stanford.edu (H.C.H.); damiencolas@gmail.com (D.C.)

**Keywords:** circadian, suprachiasmatic, memory, hippocampus, EEG, theta oscillation

## Abstract

This study examined whether theta oscillations were compromised by the type of circadian disruption that impairs hippocampal-dependent memory processes. In prior studies on Siberian hamsters, we developed a one-time light treatment that eliminated circadian timing in the central pacemaker, the suprachiasmatic nucleus (SCN). These arrhythmic animals had impaired hippocampal-dependent memory whereas animals made arrhythmic with SCN lesions did not. The current study examined whether theta oscillations are compromised by the same light treatment that produced memory impairments in these animals. We found that both methods of inducing circadian-arrhythmia shortened theta episodes in the EEG by nearly 50%. SCN-lesioned animals, however, exhibited a 3-fold increase in the number of theta episodes and more than doubled the total time that theta dominated the EEG compared to SCN-intact circadian-arrhythmic animals. Video tracking showed that changes in theta were paralleled by similar changes in exploration behavior. These results suggest that the circadian-arrhythmic SCN interferes with hippocampal memory encoding by fragmenting theta oscillations. SCN-lesioned animals can, however, compensate for the shortened theta episodes by increasing their frequency. Implications for rhythm coherence and theta sequence models of memory formation are discussed.

## 1. Introduction

Theta oscillations are critical for memory processing. They arise from the coordinated firing patterns of neuronal ensembles within brain structures and across brain regions. This synchronized activity allows information stored in discrete brain regions to be shared and processed as coherent memories [1]. Theta rhythms in the rodent electroencephalogram (EEG) oscillate at a frequency typically between 5–12 Hz, but the exact frequency is influenced by several factors such as species [2], time of day [3], sleep state [4], and brain temperature [5]. These oscillations dominate EEG signals during activities that support working memory and the encoding of episodic memories, such as when animals explore novel objects or navigate their environment [1,6]. For example, optogenetic disruption of theta rhythms during development in rats produced spatial memory deficits that persisted into adulthood [7], and in humans, enhancement of theta oscillations by brief localized magnetic stimulation improved visual working memory capacity in healthy adult subjects [8].

We previously found that loss of circadian timing induced by a one-time photic treatment, led to memory impairments in object recognition and in spatial working memory [9,10,11]. In those studies, we used a spontaneous alternation task and a novel object recognition test, both of which depend on the same septohippocampal structures that generate theta oscillations [12,13,14,15,16]. Thus, the present study examined whether the circadian disruption that led to these memory deficits also compromised expression of theta in the EEG. The experiments were performed in Siberian hamsters (*Phodopus sungorus*) because, unlike mice or rats, circadian rhythms in behavior and in the suprachiasmatic nucleus (SCN) are easily eliminated by the disruptive phase shift (DPS) protocol (see methods). The DPS protocol eliminates circadian rhythms in the central clock [17], but leaves animals neurologically and genetically intact. DPS-treated arrhythmic hamsters failed tests of object recognition and of spatial working memory [9,10,11], suggesting that an arrhythmic SCN interfered with memory encoding. This idea was confirmed in a subsequent experiment in which the SCN was surgically ablated (SCNx) in animals made arrhythmic by the DPS protocol [11]. The surgery restored performance on tests of object recognition and spatial memory to the same levels as observed in control animals [11].

The markedly different effects of DPS and SCNx models of arrhythmia on memory question the validity of the SCNx model for studies of learning and memory. Unlike SCNx animals, DPS hamsters have a dysfunctional SCN that is capable of influencing other neural structures to which it projects and can itself be influenced by feedback loops returning from those structures. In that regard, the neurophysiology of circadian dysrhythmia, like that experienced by humans, can only be resolved in models that leave the SCN intact. For obvious reasons, it can’t be done in mice with genomic changes arising from deletion of the transcriptional clockwork or animals with variable degrees of severe SCN damage. We previously described a model wherein SCN dysrhythmia might interfere with memory via changes in SCN signaling to its sole projection in the limbic system, the lateral septal nucleus [9]. This structure provides input to the medial septum and, therefore, is positioned to influence theta rhythms arising from oscillations within septal-hippocampal circuitry. Given the importance of hippocampal theta oscillations in memory encoding, we investigated the impact of SCN arrhythmia on those oscillations to see if DPS hamsters exhibited changes in theta that might account for their memory deficits.

## 2. Materials and Methods

### 2.1. Animals and Housing Conditions

Siberian hamsters (*Phodopus sungorus*) were bred in the laboratory in a 16:8-h light-dark (LD) cycle (lights on at 0200 h, PST) at an ambient temperature of 22 °C. Animals were provided with cotton batting for nesting material; food (Purina chow #5015) and tap water were available ad libitum. All experimental procedures were approved by Stanford University’s Administrative Panel on Laboratory Animal Care (Animal Use Protocol #14988) and were conducted in accordance with the NIH Guide for the Care and Use of Laboratory Animals.

Housing and lighting conditions were as described previously [10]. Prior to the start of an experiment, animals were housed individually and locomotor activity was measured by passive infrared motion detectors mounted directly above the tip of the water bottle sipper tube [10]. Activity levels primarily reflected drinking behavior and locomotor activity that occurred directly under the sipper tube. Activity bouts were summed in 10-min intervals and stored on computer. The times of day when spatial memory was tested are given by zeitgeber time (ZT) where ZT0 = time of lights-on and ZT16 = time of lights-off in the animal rooms.

### 2.2. EEG Surgery and Recording

A group of male hamsters that were 2–3 months old underwent EEG implant surgery and were allowed to recover for 4 weeks. Surgical procedures were performed according to [18] under deep anesthesia (100 mg/kg ketamine; 5 mg/kg xylazine); post-operative analgesia was achieved using meloxicam (5 mg/kg s.c). Two stainless steel screws were placed into pilot holes drilled into the skull over the frontal and parietal cortices of the right cerebral hemisphere to serve as EEG cortical electrodes. Two other screws were placed over the left hemisphere and used as anchor screws. The electrodes were soldered onto the recording leads before implantation and the anchor screws were cemented to the skull with Super-6 Bond (Sun Medical, Co., Shiga, Japan) and dental cement. Recordings were performed using an EMBLA™ amplifier and Somnologica™ software (Toronto, ON, Canada). EEG signals were amplified, passed through an analog-to-digital converter sampling at 200 Hz, and filtered based on frequency range (0.5–25 Hz).

### 2.3. Behavioral Testing: EEG

**Figure 1:**
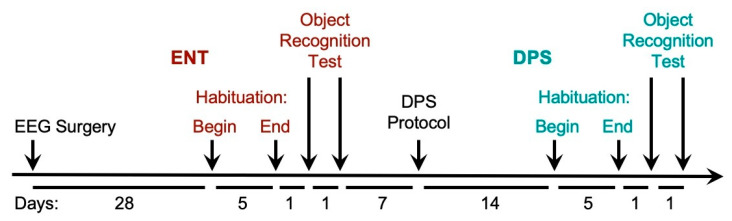
Timeline of procedures for experiment 1. Male hamsters were 2–3 months old at the time of the EEG surgery. Animals in the circadian-intact entrained (ENT, red) condition were then made circadian-arrhythmic by the disruptive phase shift (DPS, green) protocol and re-tested. The amount of time between each procedure is given in days below (timeline not to scale). Brain surgery for SCN-lesioned and Sham-operated animals was performed 4 weeks prior to the EEG surgery. Age-matched hamsters used for video tracking did not undergo surgery. For all animals, the object recognition test was conducted during the last 2 h of the day. The test consisted of 20 min of baseline EEG recording followed by 5 min of object exploration. See text for details of all procedures.

A timeline for the testing procedures is shown in Figure 1. After 4 weeks of recovery from EEG surgery, hamsters were connected to the EEG cables and placed in larger rat-sized cages (Techniplast, Inc., Montreal, QC, Canada, 33 cm × 21 cm × 18 cm) and allowed 5 days to habituate to the new cages. These cages had no lids but were fitted with elevated acrylic walls (31 cm) mounted on top of the cages to keep hamsters inside while allowing free movement during EEG recording. A thin layer of bedding (2.5 cm) was provided. Food was placed in a ceramic bowl in a corner of the cage. A water bottle attached outside the cage was fitted with a curved sipper tube that terminated inside the cage.

During the 5 days of habituation and for the remainder of the study, only the experimenter (author A.C.L.) had access to the room. In the late afternoon on each of those 5 days, the experimenter habituated the animals to her presence by interacting with them for 5 min using a gentle handling procedure. During the first 2 days of habituation, the experimenter’s hand was placed in the cage and the hamster was allowed to sniff and crawl over the hand. On days 3–5, each hamster was held in the experimenter’s hand without removing the animal from the cage. After 5 days of habituation, on the following day (day 6), the object recognition task was initiated in the late afternoon, two hours before the time of lights-off in the room. The task began by starting EEG recordings while the experimenter remained in the room at a distance of 4 feet from the cages, visually verifying that the animal was awake (i.e., eyes open, frequent locomotor movement, grooming behavior) while baseline EEG data were collected for the next 20 min.

After the 20 min, a novel object (a bright pink plastic cylinder, approximately 7.6 cm long × 3.8 cm in dia., filled with fine gravel for weight) was placed in the center of the cage parallel to the long side of the cage. Bedding was pushed aside to allow contact with the cage bottom. The EEG was recorded for an additional 5 min during which time the animal freely explored the object. The object was removed at the end of 5 min. In experiment 1, an identical copy of the object was placed in the cage 24 h later. As on the prior day, baseline EEG was recorded for 20 min, followed by object placement in the cage and 5 min of exploration.

### 2.4. Induction of Circadian Arrhythmia: The Disruptive Phase Shift (DPS) Protocol

Seven days after the object exploration test, lights in the colony room were turned on for 2 h beginning 5 h after lights-off (i.e., a 2-h light pulse from 2300 h to 0100 h). On the next day, the LD cycle was phase delayed by 3 h so that dark onset occurred 3 h later than on the previous night (i.e., time of lights-off shifted from 1800 h to 2100 h; lights-on shifted from 0200 h to 0500 h). Animals remained in the 16:8 LD cycle thereafter. Locomotor activity was continuously recorded for the next 14 days to confirm loss of circadian locomotor rhythms by chi-square periodogram analysis (ClockLab, ActiMetrics, Wilmette, IL, USA). After the 14 days of arrhythmia, hamsters that were confirmed circadian-arrhythmic by periodogram analysis were transferred to the EEG recording cages and allowed 5 days to habituate to the cages with the handling procedure. EEG recording and object exploration testing were performed as described above. Thus, a total of 26 days elapsed after the completion of testing in the ENT condition before testing was initiated in the DPS condition.

### 2.5. Behavioral Testing: Video Tracking

We used video tracking to quantify the amount of time spent exploring objects. To accomplish these recordings, we used a new cohort of animals rather than use the same animals from the EEG recordings. New animals were used to minimize the number of times an individual animal engaged in exploration of the object and to maintain the integrity of the EEG head stages. The object exploration task relies on the perceived novelty of the object. In developing this protocol, we found that over-testing reduced the hamsters’ exploratory behavior thus undermining the validity of the task. Increasing the time interval to four weeks between each test mitigated this problem, but for the present study, spacing out the recognition tasks by a full month would be problematic. It would require that each animal be tested 4 times, at which point aging might influence the animals’ behavior. In addition, the integrity of the EEG head stage could not be guaranteed over that length of time. The time frames for habituation and testing procedures, as well as for the DPS protocol, were the same as used for the EEG recordings. Video tracking was recorded through a monochrome CCD camera (Model ICD-49, Ikegami Tsushinki Co., Tokyo, Japan) mounted overhead and digitally recorded at a frame sampling rate of 30 Hz. Video data were analyzed with Ethovision XT 9.0 software (Noldus Inc., Leesburg, VA, USA) by tracking the location of an animal’s nose as it moved around the cage. The animal was considered to be exploring an object when its nose came within 1 cm of the object. Data were analyzed over 5 min of exploration for the total time of exploration, number of exploration events, and the duration of each event.

### 2.6. SCN Lesion Surgery

Animals were anesthetized with a cocktail of 100 mg/kg ketamine and 5 mg/kg xylazine. The skull was secured and held level in a stereotaxic device. An ophthalmic ointment was applied to both eyes. Two small holes were drilled in the skull and a stainless steel electrode, insulated except for 0.1 mm at the tip, was lowered into the brain targeting the SCN. Lesions were made by passing a 6 mA current for 10 s bilaterally through the tip of the electrode. Lesion coordinates are 1.0 mm anterior to bregma, ±0.2 mm lateral to the sagittal sinus, and 6.6 mm ventral to dura. The animals were administered meloxicam (5 mg/kg s.c.) as an analgesic during recovery from surgery. A control group of sham-operated animals were treated in the same manner but no current was passed through the electrode. SCN-lesioned animals (SCNx) were allowed 4 weeks to recover from surgery. Circadian-arrhythmic SCNx hamsters, as determined by periodogram analysis, then underwent surgery for EEG implants, were allowed another 4 weeks of recovery, and then moved to the EEG recording cages for 5 days of habituation followed by one day of EEG recording and object exploration.

### 2.7. Lesion Verification

At the end of the experiment, brains were removed and frozen coronal sections (30 µm) cut through the area of the optic chiasm. Mounted sections were stained with cresyl violet, and the extent of the damage was assessed microscopically. Histological evaluation of tissue damage was performed by an investigator without knowledge of the corresponding behavioral data.

### 2.8. Data Analysis

The power spectra of EEG signals were subjected to a fast Fourier transformation (FFT) at 1024 Hz on 200 Hz signals and 4 s epochs. EEG power density values were obtained for consecutive 4-sec epochs in the frequency range from 0.5–25.0 Hz in increments of 0.25 Hz within frequency bands for delta (0.5–4.0 Hz), theta (5–8 Hz). Power in each 0.25 Hz frequency bin was expressed as a percentage of total power from 0–25 Hz to facilitate visualization of peak frequencies and to normalize variation across individual hamsters (i.e., relative power). Relative power density was used for statistical comparisons between groups. EEG waveform analysis was restricted to the first 5 min of exploration. Baseline EEG measures were averaged over the 20 min period prior to object placement into the cage. All group comparisons of video-recorded exploration and power densities in each frequency band were analyzed by two-way ANOVA with repeated measures for rhythm status (ENT vs. DPS) or time (day1 vs. day 2) where appropriate. Sidak’s multiple comparisons test was used for post-hoc pairwise comparisons (Prism, v. 8.4.2, GraphPad, San Diego, CA, USA).

## 3. Results

### 3.1. Induction of Circadian Arrhythmia

**Figure 2:**
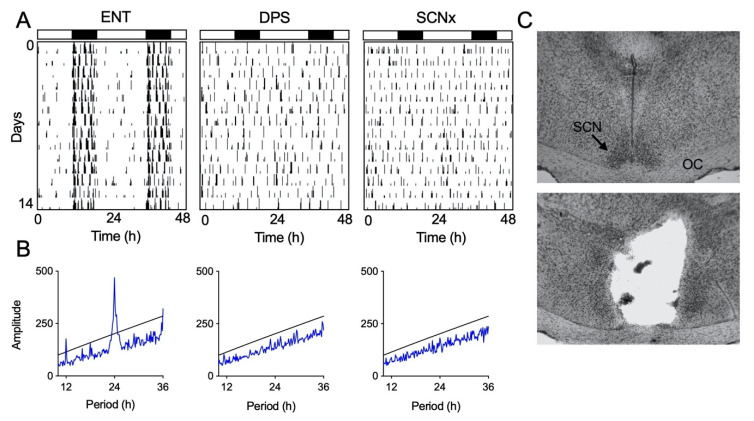
Representative examples of locomotor activity patterns. Actograms show locomotor rhythm entrainment (ENT; **A**) or circadian arrhythmia from the DPS protocol (DPS; **A**) or brain lesions (SCNx; **A**). ENT and DPS actograms are from the same animal. Vertical black hash marks indicate movement in the cage; consecutive days are double-plotted. Black and white rectangles indicate night (8 h) and day (16 h), respectively. Periodogram analysis (**B**) confirming a lack of periodicity in DPS and SCNx animals. Peaks above the black line are statistically significant (*p* < 0.001). Representative Nissl-stained sections (**C**) from intact (top) and SCNx (bottom) hamsters; optic chiasm (OC).

Locomotor activity of entrained (ENT) animals was largely restricted to the dark phase of the LD cycle (Figure 2A). Once these animals were made circadian-arrhythmic by the DPS protocol, bouts of activity occurred throughout the day and night (Figure 2A). Likewise, animals with complete bilateral ablation of the SCN (SCNx) were active throughout the day and night (Figure 2A). Periodogram analysis confirmed the presence or absence of circadian rhythms (Figure 2B).

### 3.2. Histological Analysis of Lesioned Animals

The SCN was completely bilaterally ablated in seven hamsters (SCNx). These animals also sustained damage to the paraventricular nuclei, medial preoptic and anterior hypothalamic areas. Damage to these areas ranged from 5–30%. Two hamsters that sustained damage to the optic chiasm were excluded from the final data set as a precaution because damage to visual inputs could potentially compromise their object exploration behavior. Representative tissue sections from intact and SCN-lesioned hamsters are shown in Figure 2C.

#### 3.2.1. Experiment 1. Effect of Object Exploration on the EEG of ENT and DPS Animals

**Figure 3:**
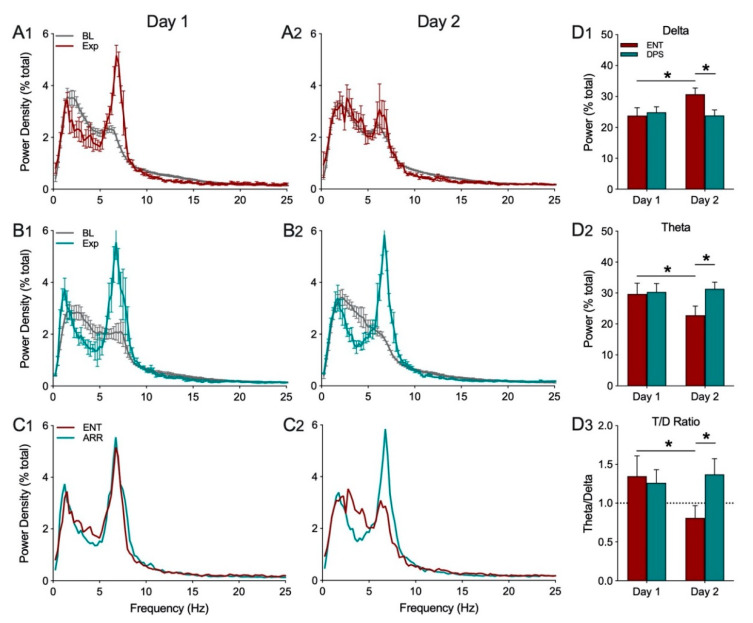
Mean (± SEM) spectral data recorded during baseline (BL, gray) and exploration under ENT (red) and DPS (green) conditions. All data were collected in the late afternoon from a single group of animals (n = 6). In the ENT condition, there was a robust decrease in EEG power in the delta range (0.5–4.0 Hz) with a corresponding increase in theta (5–8 Hz) power (**A1**) that was absent 24 h later on day 2 (**A2**). By contrast, in the DPS condition, object exploration elicited changes in both delta and theta on days 1 (**B1**) and 2 (**B2**). Spectral data during object exploration are shown without error bars to facilitate visual comparisons of the EEG under ENT and DPS conditions on days 1 (**C1**) and 2 (**C2**). Total delta power (**D1**) increased significantly, while total theta power (**D2**) decreased in ENT animals, on day 2 compared to day 1, leading to differences between ENT and DPS on day 2. A similar pattern was observed for the theta/delta (T/D) ratio (**D3**). Data expressed as mean (± SEM). * indicates significant differences; see text for *p* values.

Object exploration (Exp) decreased power in the delta frequency band (0.5–4.0 Hz), and increased power in theta (5–8 Hz), from baseline values in ENT animals on day 1, but not on day 2 (Figure 3(A1,A2)). By contrast, after ENT animals were made circadian-arrhythmic by the DPS treatment, they exhibited decreased delta and increased theta on both days 1 and 2 (Figure 3(B1,B2)). The structure of the EEG during object exploration did not differ between ENT and DPS groups on day 1, but did differ on day 2 (Figure 3(C1,C2)).

Quantitative comparisons of the effects of object exploration on total EEG power in the delta and theta frequency bands, and in the T/D ratio, revealed no significant main effects for time or rhythm status (*p* > 0.05), but did reveal significant interaction effects (delta: F_(1,5)_ = 10.93, *p* = 0.030; theta: F_(1,5)_ = 17.50, *p* = 0.014). Sidak’s post-hoc comparisons for delta showed that power increased significantly from day 1 to day 2 in the ENT condition (*p* = 0.031; Figure 3(D1)) and was significantly higher than DPS on day 2 (*p* = 0.032; Figure 3(D1)). For theta, post-hoc comparisons showed power in ENT animals decreased significantly on day 2 compared to day 1 (*p* = 0.013; Figure 3(D2)) and was significantly different compared to DPS on day 2 (*p* = 0.006; Figure 3(D2)). A similar pattern was observed for the T/D ratio. There was a significant decrease in the T/D ratio in the ENT condition from day 1 to day 2 (*p* = 0.048; Figure 3(D3)) which was also significantly higher in the DPS condition compared to ENT on day 2 (*p* = 0.041; Figure 3(D3)).

#### 3.2.2. Experiment 1. Theta-Dominated EEG Bouts

**Figure 4:**
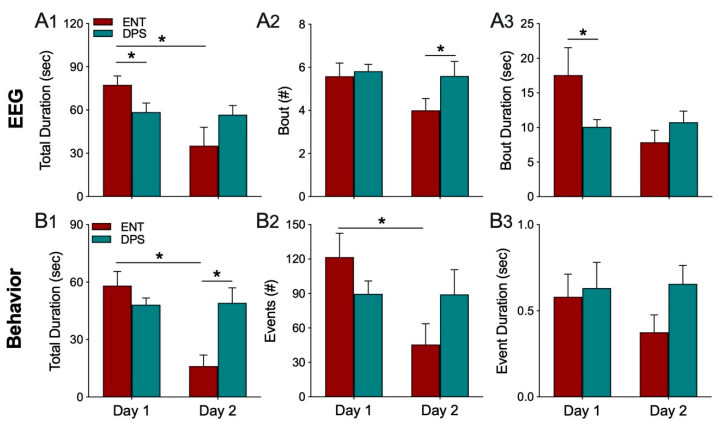
Theta dominance in the EEG (**A1**–**A3**) and exploration behavior (**B1**–**B3**) over both days of the object exploration task. In the ENT condition, both the total duration of time when EEG epochs had a T/D ratio > 1.0 (**A1**) and total time animals explored the object (**B1**) decreased significantly from day 1 to 2. The number of theta-dominated bouts (**A2**) and exploration events (**B2**) followed a similar pattern, as did the mean duration of bouts of theta (**A3**) and exploration (**B3**). By contrast, there were no changes from day 1 to 2 in any of these parameters in the DPS condition. Data expressed as mean (± SEM). * indicates significant differences (**A1**–**A3**, n = 12; **B1**–**B3**, n = 8); see text for *p* values.

EEG epochs (4 s) were analyzed to determine which epochs were theta-dominated. A theta dominated bout was defined as the number of consecutive epochs with a T/D ratio > 1.0. Thus, for example, if T/D > 1.0, for 3 consecutive bouts, the bout is 12 s (3 epochs × 4 s duration). Statistical comparisons of theta-dominated bouts suggested that animals in the DPS condition had shorter bouts of theta, but the difference was not significant. We, therefore, tested an additional six animals under the same conditions as described for the original cohort of hamsters and combined the data from both cohorts (n = 12 total; Figure 4).

The total duration of time when the EEG was dominated by theta was significantly affected by circadian rhythm status (ENT vs. DPS; F_(1,22)_ = 6.84, *p* = 0.020) and time (Day 1 vs. Day 2; F_(1,22)_ = 6.30, *p* = 0.024). Total duration in the ENT condition was significantly greater than it was for the DPS condition on day 1 (Sidak’s comparison, *p* = 0.015; Figure 4(A1)), but then decreased significantly on day 2 (Sidak’s, *p* = 0.024; Figure 4(A1)). By contrast, analysis of the number of theta-dominated bouts revealed an effect of rhythm status (F_(1,22)_ = 5.72, *p* = 0.038), but not of time (*p* > 0.05). On day 2, the number of theta-dominated bouts was significantly lower in ENT condition compared to DPS (Sidak’s, *p* = 0.016; Figure 4(A2)). Similarly, theta-dominated bout duration was significantly affected by rhythm status (F_(1,22)_ = 4.93, *p* = 0.042), but not by time (*p* > 0.05). Bout duration on day 1 was shortened by nearly half after the DPS treatment (Sidak’s, *p* = 0.034; Figure 4(A3)), but ENT and DPS groups did not differ on day 2 (*p* > 0.05).

#### 3.2.3. Experiment 1. Object Exploration Behavior

Overall, changes in theta dominance in the EEG were paralleled by changes in exploratory behavior. The total duration of time spent exploring the object was influenced by rhythm status (F_(1,14)_ = 13.15, *p* = 0.004) and time (F_(1,14)_ = 11.95, *p* = 0.005). Total duration decreased significantly from day 1 to 2 in the ENT condition (Sidak’s, *p* = 0.007), but did not decrease in the DPS condition (*p* > 0.05; Figure 4(B1)). On day 2, total duration was significantly lower in ENT compared to DPS (Sidak’s, *p* = 0.003). A similar pattern was observed in the number of exploratory events that decreased significantly across days in the ENT condition (F_(1,14)_ = 12.90, *p* = 0.012; Sidak’s, *p* = 0.011), but not in DPS (*p* > 0.05; Figure 4(B2)). By contrast, there were no statistically significant changes for the duration of exploratory events (*p* > 0.05; Figure 4(B3)).

### 3.3. Experiment 2. Theta Responses in SCNx Animals

The results of experiment 1 suggested that the DPS treatment fragmented theta bouts to such an extent that it interfered with memory encoding on day 1. The goal of experiment 2 was to determine whether an intact, albeit arrhythmic, SCN is necessary to maintain theta fragmentation. In past studies of object recognition [11], DPS and SCNx hamsters spent the same amount of time exploring an object on day 1 of an object recognition task, which is the memory encoding phase of the task [11]. SCNx hamsters, however, do not exhibit any of the memory deficits characteristic of DPS animals [11]. We hypothesized that SCNx animals would not exhibit the same theta fragmentation observed in DPS animals, therefore, we tested groups of SCNx (n = 5) and Sham-operated (Sham, n = 3) hamsters for theta fragmentation on day 1 of object exploration.

**Figure 5:**
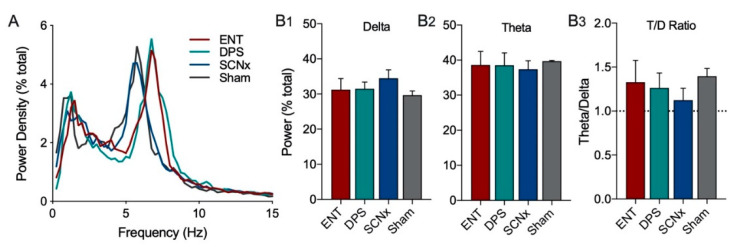
SCN lesions had no effect on total power in delta and theta bands during one day of object exploration. Power spectrum during exploration was similar among SCNx (n = 5) and Sham (n = 3) animals compared to ENT (n = 12) and DPS (n = 12) groups (**A**), however, peak theta frequency was ~1.0 Hz slower; for this reason, the theta band was defined as 4–7 Hz for SCNx and Sham groups. Total power in the delta (**B1**) and theta (**B2**) frequency bands, as well as in the T/D ratio (**B3**) did not differ among all four groups. Data expressed as mean (±SEM).

The EEG power density for ENT, DPS, SCNx, and Sham groups during object exploration did not differ in power in the delta or theta frequency bands (Figure 5A). One notable observation was that the peak theta frequency did not differ among SCNx and Sham groups, but both of those groups were ~1.0 Hz slower than ENT and DPS groups (Figure 5A), thus, we defined the theta band as 4–7 Hz for SCNx and Sham animals. It should be noted that the placement of EEG electrodes in the skull was the same for all four groups. The total amount of power did not differ (*p* > 0.05) among these four groups in delta (Figure 5(B1)), theta (Figure 5(B2)), or in the T/D ratio (Figure 5(B3)).

**Figure 6:**
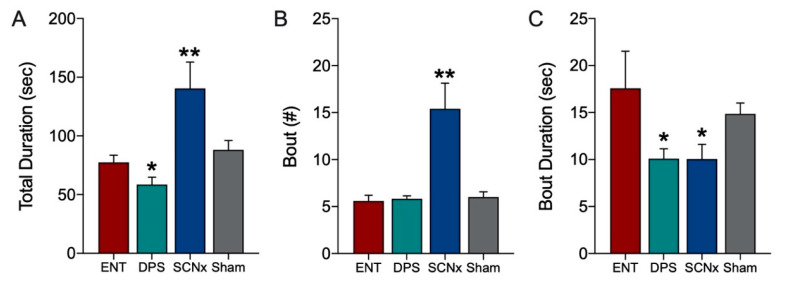
Theta dominance in the EEG during object exploration was substantially increased by SCN lesions. The total duration of time spent with an object (**A**) and the number of theta-dominated bouts (**B**) was significantly higher in SCNx compared to ENT animals. By contrast the duration of individual theta bouts (**C**) was significantly lower. Data from Sham animals are shown for comparison. Data expressed as mean (± SEM). * indicates significant differences; see text for *p* values. ** *p* < 0.001.

Theta-dominated bouts in the EEG were significantly altered by the loss of circadian timing. Because statistical comparisons among ENT and DPS conditions were already reported above (Figure 4), they were not repeated here. Means of ENT and SCNx groups were compared by *t*-tests (two-tailed). During object exploration, SCNx animals spent significantly more time in theta during exploration than did ENT animals (*p* = 0.002; Figure 6A) and had nearly 3-fold more theta-dominated bouts than did ENT animals (*p* < 0.001; Figure 6B). However, the mean duration of individual theta bouts in SCNx animals were significantly shorter than those of ENT controls (*p* = 0.025; Figure 6C).

## 4. Discussion

Theta oscillations serve a critical role in episodic and spatial memory formation, therefore, we examined theta rhythms during memory encoding to determine whether these oscillations might explain how circadian arrhythmia impairs memory in DPS hamsters. Circadian rhythms were eliminated by two methods, the DPS protocol which leaves the animals neurologically and genetically intact, and by surgical ablation of the SCN, the master circadian clock. Neither method had any effect on the EEG power spectra during baseline recordings. Likewise, the effects of object exploration on delta and theta did not differ among groups on the first day of exploration; overall, power in delta was suppressed while theta dominated the power spectra. Unlike control (ENT) animals, DPS-treated hamsters exhibited high levels of theta on both days one and two, indicating a failure to remember the object. This memory deficit was not simply due to inadequate object exploration because hamsters interacted with the objects to the same extent in both ENT and DPS conditions. Although the average duration of each interaction with the object was the same in both conditions, the duration of theta episodes was nearly 50% shorter after the DPS treatment. We propose that theta fragmentation is a cause of the memory deficits that characterize DPS hamsters.

The behavioral data provided an important insight into the relationship between exploration and theta-driven information processing. In both the ENT and DPS conditions, the mean duration of a single interaction with the object was <1.0 s while the average theta bout was >10 s for both groups. This suggests that theta oscillations continue to process information when the animal is no longer engaged with the object. Animals were only considered to be actively exploring when their nose was within 1 cm of the object, as is standard practice. But as our data show, this measure does not relate quantitatively to memory encoding. In a previous study on novel object recognition (NOR) in ENT hamsters, we tested animals at six different times of day [9]. NOR performance varied widely across the day, but the total time spent exploring objects did not change over those time points [9]. Furthermore, DPS animals spent the same amount of time exploring objects as did ENT hamsters [9,10,11], and yet, DPS animals fail to recognize a familiar object. Thus, while it is useful to operationalize behavioral exploration for comparative purposes, the amount of exploration time is not a proxy for memory encoding.

The duration of individual theta bouts rather than total time spent in theta appears to be a better predictor of memory deficits. Theta dominated the EEG of DPS animals during exploration for an average of 58 s which was only 18 s less than in the ENT condition, thus, DPS animals were able to generate a sufficient amount to theta to encode memory. The fact that DPS animals failed to recognize the object on day 2 suggests that the quality, rather than quantity, of theta is more important for encoding. In this regard, the shortening of theta oscillations, or theta fragmentation, appears to be a consequence of circadian arrhythmia whether caused by the DPS treatment or by SCN lesions. SCNx hamsters were, however, able to compensate for theta fragmentation by increasing their cognitive effort through additional theta bouts. The increased frequency of these bouts might explain why SCNx animals do not exhibit the memory deficits found in DPS hamsters; shortening the intervals between bouts (i.e., shortened temporal spacing of theta fragments) might allow greater continuity in encoding object information. Alternatively, more frequent theta bouts might increase the repetition of information carried in fragmented theta sequences.

The data from day 2 of exploration confirmed that the decrease in theta in the ENT condition was due to reduced exploration of the object. This was important to confirm because theta can be elicited in the EEG by ambulation so any observed decreases in theta could potentially be due to a general decrease in locomotor activity, but that was not the case here; the reduction in theta was associated with a reduction in exploratory behavior. We did not perform similar tests with SCNx animals because their purpose was to determine whether arrhythmia by means different from DPS would produce similar results in theta fragmentation. It is likely that their behavior on day 2 would have been similar to the ENT condition given that SCNx hamsters exhibit the same day-2 reduction in time spent with a familiar object as do ENT animals in a 2-day NOR task [11].

Given the importance of theta rhythms in memory, it is worth examining the implications of the present findings for models of memory involving neuronal synchrony and acetylcholine signaling. Theta bout fragmentation could impair memory through its effect on acetylcholine (Ach) release in hippocampal circuits. Ach signaling supports spatial working memory [13,14,15] via its release from medial septal neurons onto cells in the dentate gyrus and CA1 [12,15]. This phasic release of Ach from septal neurons is highly correlated with theta frequency [19,20]. Furthermore, optogenetic activation of septal cholinergic neurons enhances theta oscillations in hippocampal neurons via a mechanism that appears to involve activation of inhibitory interneurons, as well as inhibition of principal neurons, in the hippocampus [21,22]. Anatomical evidence from mice, rats, and hamsters indicates that the SCN could influence both theta and Ach release through its projection to the ventrolateral septal nuclei [23,24,25], which gates the expression of theta frequency [26] and innervates the medial septum (MS) [27]. The MS is a major source of Ach in the brain, thus, the SCN is well positioned to influence cholinergic modulation of theta via the septohippocampal pathway and by additional cholinergic projections to other regions of the hippocampus.

Circadian arrhythmia in the SCN could interfere with hippocampal mechanisms of memory processing by disruption of synchronized firing among neuronal populations. Synchronous neuronal activity is a critical feature of memory formation whether it occurs within a neuronal ensemble or across distant brain regions [28,29]. At the heart of many rhythm coherence models are theta oscillations that provide the timing cue for synchronous phase-specific firing in phase precession of hippocampus place cells or long-range synchrony between the hippocampus and medial prefrontal cortex (mPFC) [1,29,30]. Numerous studies have established that reduced synchrony among cells or brain regions impairs the memory functions subserved by those neuronal populations (for review see [29]). During memory tasks, for example, neurons in the mPFC synchronize to theta oscillations [31,32], with greater synchrony occurring during correct learning trials compared to incorrect ones [31,33]. Thus, the mechanism by which shortened theta bouts could impair memory might involve disruptions in synchrony among neuronal ensembles. Alternatively, the disruptions in theta reported here might impair memory by breaking up, or truncating, chunks of information that are carried within each theta sequence [34,35]. In rats trained on a rewarded spatial path decision task [34], the encoding of path length was directly related to the length of the theta cycle. This suggests that theta bouts shortened (i.e., fragmented) by circadian arrhythmia would result in the misrepresentation of spatial information encoded in memory and, perhaps, in the erroneous recall of spatial representations.

The DPS model has revealed a previously unknown role of the SCN in memory encoding via its effects on theta oscillations. The present study shows that the memory impairments observed in animals made circadian-arrhythmic by the DPS protocol require an active, albeit arrhythmic, SCN to maintain those impairments. The SCN is a relatively small structure with a sparse projection to the ventrolateral septal nuclei [23,24,25], its sole projection to the limbic system. And yet, dysfunction within the SCN is able to disrupt theta oscillations that arise from several major structures in the septal-hippocampal system. Remarkably, the arrhythmic SCN continues to maintain memory impairments even when 60% of the nucleus is surgically ablated [11]. Circadian disruption in SCN-intact rats and mice has long been known to impair hippocampal-dependent memory [36], but the neural mechanisms that link the SCN to the hippocampus are largely unknown. Our data suggest that the SCN impacts the anatomically distant hippocampus by its influence over theta oscillations.
